# A tale of two countries: all‐cause mortality among people living with HIV and receiving combination antiretroviral therapy in the UK and Canada

**DOI:** 10.1111/hiv.12505

**Published:** 2017-04-24

**Authors:** S Patterson, S Jose, H Samji, A Cescon, E Ding, J Zhu, J Anderson, AN Burchell, C Cooper, T Hill, M Hull, MB Klein, M Loutfy, F Martin, N Machouf, JSG Montaner, M Nelson, J Raboud, SB Rourke, C Tsoukas, RS Hogg, C Sabin, the Canadian Observational Cohort (CANOC) ‐ UK Collaborative HIV Cohort (UK CHIC) Collaboration, Deborah Kelly, Stephen Sanche, Alexander Wong, Tony Antoniou, Ahmed Bayoumi, Bohdan Nosyk, Michelle Cotterchio, Charlie Goldsmith, Silvia Guillemi, P. Richard Harrigan, Marianne Harris, Sean Hosein, Sharon Johnston, Clare Liddy, Viviane Lima, David Moore, Alexis Palmer, Peter Phillips, Anita Rachlis, Marek Smieja, Benoit Trottier, Mark Wainberg, Sharon Walmsley, Chris Archibald, Ken Clement, Monique Doolittle‐Romas, Laurie Edmiston, Brian Huskins, Jerry Lawless, Douglas Lee, Renee Masching, Stephen Tattle, Alireza Zahirieh, Claire Allen, Stryker Calvez, Guillaume Colley, Jason Chia, Daniel Corsi, Louise Gilbert, Nada Gataric, Alia Leslie, Lucia Light, David Mackie, Costa Pexos, Susan Shurgold, Leah Szadkowski, John Wong, Benita Yip, Jaime Younger, Jonathan Ainsworth, Sris Allan, Abdel Babiker, David Chadwick, Valerie Delpech, David Dunn, Martin Fisher, Brian Gazzard, Richard Gilson, Mark Gompels, Phillip Hay, Margaret Johnson, Stephen Kegg, Clifford Leen, Chloe Orkin, Adrian Palfreeman, Andrew Phillips, Deenan Pillay, Frank Post, Jillian Pritchard, Memory Sachikonye, Achim Schwenk, Anjum Tariq, John Walsh, Alicia Thornton, Adam Glabay, Nicky Perry, Stuart Tilbury, Elaney Youssef, Duncan Churchill, Rhiannon Everett, David Asboe, Sundhiya Mandalia, Hardik Korat, Chris Taylor, Zachary Gleisner, Fowzia Ibrahim, Lucy Campbell, Nataliya Brima, Ian Williams, Mike Youle, Fiona Lampe, Colette Smith, Rob Tsintas, Clinton Chaloner, Samantha Hutchinson, Susie Huntington, Nicky Mackie, Alan Winston, Jonathan Weber, Farhan Ramzan, Mark Carder, Janet Lynch, James Hand, Carl de Souza, Sajid Munshi, Sheila Miller, Chris Wood, Alan Wilson, Sheila Morris, Sue Allan, Khurram Memon, Adam Lewszuk, Emma Cope, Jane Gibson, Paul Main, Mandip Dhillon, Sarah Russell‐Sharpe, Andrew Harte, Stephen Clay, Hazel Spencer, Ron Jones, Shirley Cumming, Claire Atkinson, Roy Trevelion

**Affiliations:** ^1^ British Columbia Centre for Excellence in HIV/AIDS Vancouver BC Canada; ^2^ Faculty of Health Sciences Simon Fraser University Burnaby BC Canada; ^3^ Research Department of Infection and Population Health University College London London UK; ^4^ British Columbia Centre for Disease Control Vancouver BC Canada; ^5^ Northern Ontario School of Medicine Sudbury ON Canada; ^6^ Homerton University Hospital NHS Trust London UK; ^7^ Department of Family and Community Medicine St Michael's Hospital Toronto ON Canada; ^8^ Li Ka Shing Knowledge Institute Toronto ON Canada; ^9^ Dalla Lana School of Public Health University of Toronto Toronto ON Canada; ^10^ The Ottawa Hospital Division of Infectious Diseases University of Ottawa Ottawa ON Canada; ^11^ Faculty of Medicine McGill University Montreal QC Canada; ^12^ The Montreal Chest Institute McGill University Health Centre Montreal QC Canada; ^13^ Faculty of Medicine University of Toronto Toronto ON Canada; ^14^ Maple Leaf Medical Clinic Toronto ON Canada; ^15^ Women's College Research Institute Toronto ON Canada; ^16^ York Teaching Hospital NHS Foundation Trust York UK; ^17^ Clinique Medicale l'Actuel Montreal QC Canada; ^18^ Faculty of Medicine University of British Columbia Vancouver British Columbia Canada; ^19^ Chelsea and Westminster Hospital NHS Trust London UK; ^20^ Toronto General Research Institute University Health Network Toronto ON Canada; ^21^ Ontario HIV Treatment Network Toronto ON Canada

**Keywords:** AIDS, antiretroviral therapy, Canada, HIV, mortality, UK

## Abstract

**Objectives:**

We sought to compare all‐cause mortality of people living with HIV and accessing care in Canada and the UK.

**Methods:**

Individuals from the Canadian Observational Cohort (CANOC) collaboration and UK Collaborative HIV Cohort (UK CHIC) study who were aged ≥ 18 years, had initiated antiretroviral therapy (ART) for the first time between 2000 and 2012 and who had acquired HIV through sexual transmission were included in the analysis. Cox regression was used to investigate the difference in mortality risk between the two cohort collaborations, accounting for loss to follow‐up as a competing risk.

**Results:**

A total of 19 960 participants were included in the analysis (CANOC, 4137; UK CHIC, 15 823). CANOC participants were more likely to be older [median age 39 years (interquartile range (IQR): 33, 46 years) *vs*. 36 years (IQR: 31, 43 years) for UK CHIC participants], to be male (86 *vs*. 73%, respectively), and to report men who have sex with men (MSM) sexual transmission risk (72 *vs*. 56%, respectively) (all *P* < 0.001). Overall, 762 deaths occurred during 98 798 person‐years (PY) of follow‐up, giving a crude mortality rate of 7.7 per 1000 PY [95% confidence interval (CI): 7.1, 8.3 per 1000 PY]. The crude mortality rates were 8.6 (95% CI: 7.4, 10.0) and 7.5 (95% CI: 6.9, 8.1) per 1000 PY among CANOC and UK CHIC study participants, respectively. No statistically significant difference in mortality risk was observed between the cohort collaborations in Cox regression accounting for loss to follow‐up as a competing risk (adjusted hazard ratio 0.86; 95% CI: 0.72–1.03).

**Conclusions:**

Despite differences in national HIV care provision and treatment guidelines, mortality risk did not differ between CANOC and UK CHIC study participants who acquired HIV through sexual transmission.

## Introduction

Canada and the UK are two high‐income countries with notable differences in health care provision for people living with HIV (PLWH). However, whether clinical prognoses differ for PLWH receiving care between these two settings remains undefined.

Ongoing health care and treatment for all 107 800 PLWH in the UK is provided free of charge through the National Health Service (NHS) 1. HIV treatment guidelines and standards of care documents published by the British HIV Association (BHIVA), together with a national service specification from NHS England, provide a universal national benchmark to guide clinical HIV care and prescribing decisions throughout the UK 2, 3.

In Canada, there are 75 500 PLWH 4, and although select provinces provide treatment guidelines, no acknowledged national standards in HIV care exist. In British Columbia (BC) and Québec, HIV treatment is formally standardized by comprehensive provincial guidelines 5, 6. Other Canadian provinces informally follow International Antiviral Society (IAS) guidelines 7 and/or recommendations from the US Department of Health and Human Services 8. Antiretroviral therapy (ART) funding mechanisms vary across Canada, ranging from complete coverage to partial coverage or income‐based reimbursement 9, 10, 11.

Recent work has shown reduced attrition at all stages of the HIV care cascade among PLWH in the UK compared with Canadian settings 12. In light of the aforementioned differences in HIV care provision between the UK and Canada, and emerging disparities in the HIV care cascade 12, we sought to compare all‐cause mortality within a subset of the populations infected with HIV via sexual transmission in these two settings. All‐cause mortality has been recognized as a preferred prognostic indicator for PLWH 13. This outcome variable was selected as a basic, easily interpreted measure of cohort health status 13.

## Methods

Subsets of data from two national cohort collaborations investigating HIV clinical outcomes and treatment responses, the Canadian Observational Cohort (CANOC) collaboration and UK Collaborative HIV Cohort (CHIC) study, were merged in September 2014. At this time, data were available from 1 January 2000 up to the end of 2012 for both cohort collaborations.

### CANOC collaboration

The CANOC collaboration is a multi‐site cohort of PLWH initiating ART for the first time after 1 January 2000, and was established to evaluate patterns of treatment uptake and response, and health service provision and outcomes across Canada. Participants must reside in Canada and be aged at least 18 years, with documented HIV infection. Each contributing cohort site performs data extraction of demographic, laboratory and clinical variables, submitted annually to the coordinating centre in Vancouver for data merging, cleaning and analysis. At the time of writing, almost 10 000 participants had contributed data from eight cohorts located within the country's most populous provinces: BC, Ontario and Québec. The BC cohort submits full population‐level data, whereas sites in Ontario and Québec largely capture clinic‐based data. Ethics board approval of the CANOC collaboration was granted to each participating cohort site. A detailed cohort profile has been published 14.

### UK CHIC study

The UK CHIC study is a collaboration that currently includes 19 participating NHS HIV treatment centres. The study was established in 2001 with the aim of evaluating treatment uptake and clinical outcomes of PLWH accessing care in the UK 15, 16. The UK CHIC study currently includes over 50 000 participants aged > 16 years, who have received HIV care at one of the collaborating centres in England and Scotland on at least one occasion since 1996. Each participating centre submits electronic data annually to the coordinating group based at University College London (UCL) and the Medical Research Council Clinical Trials Unit. The coordinating group merges de‐identified data into a final data set after performing data cleaning and quality checks. The UK CHIC study has been approved by a multi‐centre research ethics committee and by local ethics committees. Further details can be found in the cohort profile 16.

### Inclusion criteria

Participants included in this merged collaborative analysis were aged 18 years or older, initiated ART for the first time between 2000 and 2012, had complete information on gender, sexual transmission risk, CD4 cell count and HIV viral load (VL) prior to ART initiation (baseline), and had at least one follow‐up measure of CD4 cell count and VL. Participant inclusion was limited to individuals presumed infected via sexual routes to improve cohort comparability, as a consequence of the low prevalence of individuals who have acquired HIV through injecting drug use (IDU) in the UK compared with Canada (2 *vs*. 17%, respectively) 1, 17. Nonetheless, we carried out a sensitivity analysis including persons with a history of IDU to evaluate potential bias that may have been introduced as a result of their exclusion from the primary analysis.

### Measures

#### Primary outcome variable

The primary outcome of interest was all‐cause mortality, defined as date of death. In the UK CHIC study, date of death is requested as part of the annual data submission from participating centres. Additionally, records for the majority (94%) of UK CHIC participants are linked to National HIV Surveillance data at Public Health England to supplement information on deaths. These data sets also receive mortality information from the Office of National Statistics for deaths occurring under the age of 65 years in the UK. The completeness of death ascertainment among UK CHIC study participants is therefore high. In CANOC, mortality ascertainment varies within participating cohorts. Two of the eight participating cohorts have established linkages to vital statistics registries, allowing for excellent ascertainment of deaths. However, other participating cohorts submit date of death as part of regular data submission to the coordinating centre, with no linkage to vital statistics, and consequently death ascertainment is less complete.

#### Explanatory variables of interest

Explanatory variables included were age at ART initiation (per decade), sexual transmission risk category [men who have sex with men (MSM), heterosexual male or heterosexual female], ethnicity (Caucasian, Black, Asian, other or unknown), hepatitis C serostatus, defined as hepatitis C antibody positive at treatment initiation (yes, no or unknown), baseline diagnosis of AIDS‐defining illness (ADI) (yes or no), baseline CD4 cell count (per 100 cells/μL) and HIV RNA plasma VL (log_10_ HIV‐1 RNA copies/mL), composition of initial antiretroviral regimen, and era of ART initiation (2000–2003, 2004–2007 or 2008–2012).

### Loss to follow‐up

Cohort studies often fail to accurately classify mortality outcomes among persons lost to follow‐up, resulting in under‐ascertainment of deaths and affecting study validity 18. Mortality among persons lost to follow‐up is reported to range from 28 to 40% 19, 20, 21, 22, 23, 24. Other cohort collaborations have noted considerable variation in the attrition of cohort participants; the Antiretroviral Therapy Cohort Collaboration (ART‐CC) reported that loss to follow‐up varied between participating cohorts from 2 to 18% 19. We defined loss to follow‐up as no clinical contact for at least 18 months, and accounted for this important variable as a competing risk in our statistical analysis. Competing risk analyses maintain participants in the analysis who are lost to follow‐up among those who are at risk of dying, rather than censoring them at the last point of clinical contact 25.

#### Statistical models

Differences in baseline sociodemographic and clinical characteristics between cohort collaborations were evaluated using chi‐square or Fisher's exact test for categorical variables and the Wilcoxon rank sum test for continuous variables. Crude mortality rates were calculated per 1000 person‐years (PY) by dividing total deaths by total person‐years of follow‐up.

Competing risks Cox regression 25 identified variables associated with mortality during study follow‐up, accounting for loss to follow‐up as a competing risk. Previous work has shown that all‐cause mortality and causes of death differ with time since ART initiation, with all‐cause mortality highest in the first year following ART initiation, largely as a result of AIDS‐related deaths 26, 27. We therefore compared mortality rates between the cohort collaborations within the first year since ART initiation as well as for the entire follow‐up period. Variables considered likely to have an impact on the risk of death following literature review and clinical hypothesis were *a priori* candidates for model inclusion. Due to the large proportion of missing data for ethnicity and hepatitis C antibody status, these covariates were not included in the adjusted analysis. An exploratory model selection process based on the Akaike information criterion and type III *P*‐values was used to guide final model selection. Statistical tests were considered significant at *α* = 0.05. Analyses were conducted using sas 9.4 software (SAS Institute, Cary, NC, USA).

#### Sensitivity analysis

Individuals who acquired HIV through IDU represent 17% of the Canadian population living with HIV 1, 17. As these individuals were excluded, CANOC participants included in the main analysis did not fully represent the Canadian population living with HIV. Previous work has shown that Canadians with a history of IDU have increased all‐cause mortality rates 28, 29; thus, we were aware that exclusion of injecting drug users from our main analysis would underestimate the mortality rate within CANOC. To generate crude mortality estimates more accurately representing the Canadian HIV epidemic, we performed a sensitivity analysis within an analytic sample that included participants reporting IDU transmission risk from each cohort collaboration.

## Results

Of the 19 960 individuals included in the main analysis, 4137 were CANOC and 15 823 were UK CHIC study participants. CANOC participants were older [median 39 years (interquartile range (IQR) 33, 46 years) *vs*. 36 years (IQR: 31, 43 years) for UK CHIC participants], and were more likely to be male (86 *vs*. 73%, respectively) and to report MSM sexual transmission risk (72 *vs*. 56%, respectively) (all *P* < 0.001) (Table 1).

**Table 1:** Comparison of sociodemographic and clinical characteristics for the Canadian Observational Cohort (CANOC) and UK Collaborative HIV Cohort (UK CHIC) study (*n* = 19 960)

Characteristic	Category	CANOC and UK CHIC *n* (%) or median (IQR)	CANOC (*n* = 4137) *n* (%) or median (IQR)	UK CHIC (*n* = 15 823) *n* (%) or median (IQR)	*P*‐value
Gender	Male	15072 (76)	3572 (86)	11500 (73)	< 0.001
	Female	4888 (24)	565 (14)	4323 (27)	
Age at ART initiation (years)	–	37 (31,44)	39 (33,46)	36 (31,43)	< 0.001
HIV sexual transmission risk	MSM	11842 (59)	2985 (72)	8857 (56)	< 0.001
	Heterosexual male	3230 (16)	587 (14)	2643 (17)	
	Heterosexual female	4888 (24)	565 (14)	4323 (27)	
Ethnicity	Caucasian	9999 (50)	1569 (38)	8430 (53)	< 0.001
	Black	6272 (31)	547 (13)	5725 (36)	
	Asian	713 (4)	202 (5)	511 (3)	
	Mixed	689 (3)	93 (2)	596 (4)	
	Other	846 (4)	285 (7)	561 (4)	
	Unknown	1441 (7)	1441 (35)	0	
Hepatitis C virus positive	No	10578 (53)	748 (18)	9830 (62)	< 0.001
	Yes	546 (3)	129 (3)	417 (3)	
	Unknown	8836 (44)	3260 (79)	5576 (35)	
Baseline CD4 count (cells/μL)	–	220 (120,318)	230 (120,328)	219 (120, 314)	0.007
Baseline VL (log_10_ copies/mL)	–	5 (4, 5)	5 (4, 5)	5 (4, 5)	0.06
ADI at baseline	No	17040 (85)	4365 (84)	13575 (86)	0.001
	Yes	2920 (15)	672 (16)	2248 (14)	
Third drug class in first regimen	NNRTI	12453 (62)	1858 (45)	10595 (67)	< 0.001
	Single PI	718 (4)	255 (6)	463 (3)	
	Boosted PI	5395 (27)	1753 (42)	3642 (23)	
	NRTI	303 (2)	84 (2)	219 (1)	
	Other	1091 (5)	187 (5)	904 (6)	
Third drug in first regimen	Efavirenz	10148 (51)	1478 (36)	8670 (55)	< 0.001
	Nevirapine	2136 (11)	309 (7)	1827 (12)	
	Lopinavir	2284 (11)	618 (15)	1666 (11)	
	Atazanavir	1997 (10)	902 (22)	1095 (7)	
	Nelfinavir	346 (2)	148 (4)	198 (1)	
	Saquinavir	340 (2)	53 (1)	287 (2)	
	Other	1091 (14)	629 (15)	2080 (13)	
NRTI backbone of first regimen	Tenofovir/emtricitabine	9262 (46)	1762 (43)	7500 (47)	< 0.001
	Zidovudine/lamivudine	4550 (23)	938 (23)	3612 (23)	
	Tenofovir/lamivudine	793 (4)	257 (6)	536 (3)	
	Abacavir/lamivudine	2719 (14)	677 (16)	2042 (13)	
	Stavudine/lamivudine	579 (3)	291 (7)	288 (2)	
	Other	2057 (10)	212 (5)	1845 (12)	
Era ART initiated	2000–2003	4827 (24)	1021 (25)	3806 (24)	0.003
	2004–2007	6540 (33)	1266 (31)	5274 (33)	
	2008–2012	8593 (43)	1850 (45)	6743 (43)	
Lost to follow‐up^a^	Yes	2548 (13)	556 (13)	1992 (13)	0.1
	No	17412 (87)	3581 (87)	13831 (87)	
Died during follow‐up	Yes	762 (4)	179 (4)	583 (4)	0.06
	No	19198 (96)	3958 (96)	15240 (96)	
Total years of follow‐up	–	4 (2, 8)	4 (2, 8)	4 (2, 8)	0.30

ADI, AIDS‐defining illness; VL, viral load; ART, antiretroviral therapy; IQR, interquartile range; MSM, men who have sex with men; NNRTI, nonnucleoside reverse transcriptase inhibitor; PI, protease inhibitor; NRTI, nucleoside reverse transcriptase inhibitor.

Column totals may not consistently add up to 100% because of rounding.

a Loss to follow‐up is defined as no clinical contact for ≥ 18 months.

**Table 2:** Competing risk Cox regression of time to death (a) during entire follow‐up period, and (b) in the year after antiretroviral therapy (ART) initiation, with loss to follow‐up as a competing risk (*n* = 19 960)

	Unadjusted HR (95% CI)	Adjusted HR (95% CI)
(a)		
Cohort Collaboration		
UK CHIC	1.00	1.00
CANOC	1.15 (0.98, 1.36)	0.86 (0.72, 1.03)
Age at ART initiation (per decade)	1.69 (1.59, 1.80)	1.67 (1.56, 1.78)
Sexual transmission risk		
MSM	1.00	1.00
Heterosexual male	1.50 (1.26, 1.79)	1.15 (0.96, 1.39)
Heterosexual female	0.88 (0.73, 1.10)	0.97 (0.80, 1.18)
Baseline CD4 count (per 100 cells/μL)	0.78 (0.73, 0.84)	0.84 (0.78, 0.91)
Baseline VL (log_10_ copies/mL)	1.53 (1.28, 1.84)	1.17 (0.97, 1.42)
Third drug in first ART regimen		
Efavirenz	1.00	1.00
Lopinavir	1.36 (1.10, 1.69)	1.36 (1.09, 1.69)
Nevirapine	1.08 (0.87, 1.34)	1.13 (0.90, 1.42)
Atazanavir	1.08 (0.81, 1.44)	1.22 (0.89, 1.66)
Other	1.46 (1.21, 1.76)	1.45 (1.18, 1.77)
Backbone of first ART regimen		
Tenofovir/emtricitabine	1.00	1.00
Zidovudine/lamivudine	1.43 (1.17, 1.75)	1.36 (1.03, 1.81)
Abacavir/lamivudine	1.48 (1.15, 1.90)	1.43 (1.08, 1.90)
Other	2.04 (1.67, 2.49)	1.64 (1.24, 2.16)
Era of ART initiation		
2000–2003	1.00	1.00
2004–2007	0.78 (0.66, 0.91)	0.88 (0.72, 1.07)
2008–2012	0.57 (0.46, 0.70)	0.84 (0.62, 1.15)
(b)		
Cohort Collaboration		
UK CHIC	1.00	1.00
CANOC	0.97 (0.7, 1.35)	0.71 (0.50, 1.01)
Age at ART initiation (per decade)	1.62 (1.44, 1.82)	1.51 (1.34, 1.71)
Sexual transmission risk		
MSM	1.00	1.00
Heterosexual male	2.08 (1.53, 2.83)	1.35 (0.97, 1.86)
Heterosexual female	0.96 (0.68, 1.36)	0.85 (0.59, 1.22)
Baseline CD4 count (per 100 cells/μL)	0.61 (0.54, 0.69)	0.65 (0.57, 0.74)
Baseline VL (log_10_ copies/mL)	1.53 (1.13, 2.06)	1.04 (0.78, 1.39)
Third drug in first ART regimen		
Efavirenz	1.00	1.00
Lopinavir	1.83 (1.24, 2.69)	1.81 (1.22, 2.7)
Nevirapine	0.68 (0.38, 1.22)	0.73 (0.4, 1.32)
Atazanavir	1.36 (0.84, 2.19)	1.5 (0.9, 2.49)
Other	2.45 (1.78, 3.39)	2.66 (1.89, 3.73)
Backbone of first ART regimen		
Tenofovir and emtricitabine	1.00	1.00
Zidovudine and lamivudine	1.08 (0.75, 1.56)	1.37 (0.85, 2.21)
Abacavir and lamivudine	1.61 (1.09, 2.38)	1.72 (1.13, 2.63)
Other	1.8 (1.28, 2.53)	1.55 (0.99, 2.41)
Era of ART initiation		
2000–2003	1.00	1.00
2004–2007	1.02 (0.74, 1.42)	1.17 (0.79, 1.72)
2008–2012	0.83 (0.59, 1.17)	1.32 (0.82, 2.14)

ADI, AIDS‐defining illness; CI, confidence interval; HR, hazard ratio; VL, viral load; MSM, men who have sex with men; NNRTI, nonnucleoside reverse transcriptase inhibitor; PI, protease inhibitor; CANOC, Canadian Observational Cohort; UK CHIC, UK Collaborative HIV Cohort.

### Clinical profile

Median baseline CD4 cell count was low across both the CANOC collaboration and UK CHIC study [230 (IQR: 120, 328) and 219 (IQR: 120, 314) cells/μL, respectively; *P* = 0.007] (Table 1). When evaluated across the three eras of ART initiation (2000–2003, 2004–2007 and 2008–2012), median baseline CD4 count was found to increase with more recent ART era within the CANOC collaboration [174 (IQR: 80, 263), 193 (IQR: 107, 270) and 270 (IQR: 170, 357) cells/μL, respectively] and the UK CHIC study [170 (IQR: 70, 271), 198 (IQR: 109, 266) and 288 (IQR: 180, 390) cells/μL, respectively] (both *P* < 0.001).

### Treatment profile

Initial ART regimen varied significantly between the cohort collaborations. A larger proportion of UK CHIC participants initiated a nonnucleoside reverse transcriptase inhibitor (NNRTI) as the third drug class within the initial regimen compared with CANOC participants (67 *vs*. 45%, respectively). CANOC participants were significantly more likely to initiate a regimen including a boosted protease inhibitor (PI) (42 *vs*. 23% in UK CHIC) (*P* < 0.001). For both CANOC and UK CHIC participants, efavirenz was most commonly used as the third antiretroviral agent in the primary regimen (36 *vs*. 55%, respectively); however, CANOC participants were more likely than UK CHIC participants to be prescribed atazanavir (22 *vs*. 7%, respectively) (*P* < 0.001). Era of ART initiation differed slightly between cohort collaborations, with a greater proportion of UK CHIC participants initiating ART in the era 2004–2007 compared with CANOC participants (33 *vs*. 31%, respectively; *P* = 0.003) (Table 1).

### Loss to follow‐up

In total, 2548 participants were lost to follow‐up over a period of 98 798 PY, to give an attrition rate of 25.8 [95% confidence interval (CI) 24.8, 26.8] per 1000 PY. When stratified by cohort collaboration, the attrition rates were 26.9 (95% CI: 24.7, 29.2) and 25.5 (95% CI: 24.4, 26.7) per 1000 PY for CANOC and the UK CHIC study, respectively.

### Mortality during total follow‐up period

**Figure 1:**
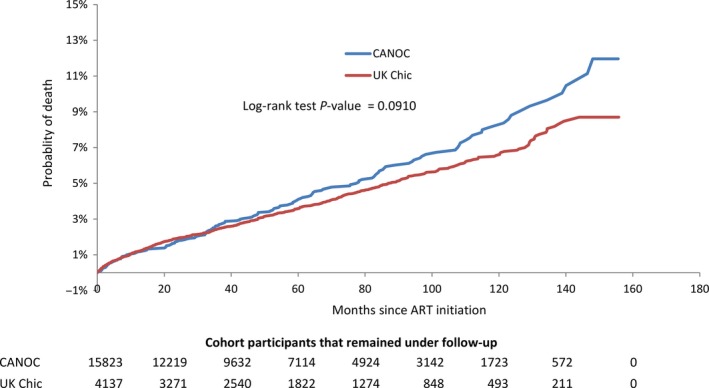
Kaplan−Meier plot showing time to death stratified by cohort collaboration (*n* = 19 960). ART, antiretroviral therapy; CANOC, Canadian Observational Cohort; UK CHIC, UK Collaborative HIV Cohort.

There were 762 deaths during a total follow‐up time of 98 798 PY, giving a crude mortality rate of 7.7 (95% CI: 7.1, 8.3) per 1000 PY. When stratified by cohort collaboration, the mortality rate was 8.6 (95% CI: 7.4, 10.0) per 1000 PY among CANOC participants, and 7.5 (95% CI: 6.9, 8.1) per 1000 PY among UK CHIC participants (Figure 1). In the competing risk survival analysis, no statistically significant difference in mortality was observed between the cohort collaborations after adjusting for participant age, era of ART initiation, sexual transmission risk category, CD4 cell count and VL at treatment initiation, and initial ART regimen [adjusted hazard ratio (AHR) 0.86; 95% CI: 0.72, 1.03] (Table 2a). Notably, the direction of the cohort effect on mortality was seen to reverse in the adjusted analysis, which was largely attributed to adjustment for participant age.

### Mortality in the year after ART initiation

There were 220 deaths during the first year after ART initiation, giving a crude mortality rate of 2.2 (95% CI: 1.9, 2.5) per 1000 PY. When stratified by cohort collaboration, the mortality rate within the first year after ART initiation was 2.2 (95% CI: 1.6, 2.9) per 1000 PY among CANOC participants, and 2.2 (95% CI: 1.9, 2.6) per 1000 PY among UK CHIC participants. In competing risk survival analysis there was no statistically significant difference in mortality in the year after ART initiation between CANOC and UK CHIC participants after adjusting for confounders (AHR 0.71; 95% CI: 0.50, 1.01) (Table 2b).

### Sensitivity analysis

When we included participants with IDU transmission risk, 2282 participants were added to the original analytic sample: 1934 additional CANOC participants and 348 additional UK CHIC participants (see Supporting Information Tables). There were 1161 deaths during a total follow‐up time of 110 414 PY, producing a crude mortality rate of 10.5 (95% CI: 9.9, 11.1) per 1000 PY. When stratified by cohort collaboration, the mortality rate was 17.0 (95% CI: 15.6, 18.6) per 1000 PY among CANOC participants, and 8.0 (95% CI: 7.4, 8.7) per 1000 PY among UK CHIC participants. In a competing risk survival analysis, no significant difference in mortality was observed between the cohort collaborations (AHR 0.9; 95% CI: 0.8, 1.1) after adjusting for confounders (see Supporting Information Tables).

## Discussion

This analysis presents a novel comparison of sociodemographic and clinical profiles and mortality outcomes for individuals living with HIV and accessing ART within two high‐income settings: Canada and the UK. Despite differences in national HIV care provision, treatment guidelines, and clinical characteristics, mortality risk did not differ significantly between CANOC and UK CHIC study participants in Cox regression accounting for loss to follow‐up as a competing risk.

### Baseline clinical profile

No clinically relevant differences in baseline CD4 cell count, VL and ADI prevalence were observed between CANOC and UK CHIC study participants within our main analysis. We hypothesized that differences were probably minimized by limiting our analysis to participants acquiring HIV through sexual transmission. However, the baseline clinical profile across cohort collaborations remained largely unchanged when participants reporting HIV acquisition through IDU were included within a sensitivity analysis. Within both cohort collaborations, CD4 cell count at treatment initiation, an important indicator of timing of treatment initiation, increased significantly with more recent treatment initiation era, reflecting evolving treatment guidelines over the period of analysis 2, 30, 31, 32.

### Treatment profile

Initial ART regimens differed between CANOC and UK CHIC participants, with CANOC participants being more likely to initiate boosted PI‐based regimens compared with UK CHIC participants. Both the ‘third’ drug in the initial treatment regimen and the backbone regimen also varied between cohort collaborations. While current treatment guidelines followed in UK and Canadian settings are broadly consistent, recommending two nucleoside reverse transcriptase inhibitors (NRTIs) plus an NNRTI or boosted PI as the initial third agent, the specific antiretroviral recommendations vary 2, 7, 8. As well as being influenced by treatment guidelines, prescribing patterns may be based on the availability of regimens and drug costs across different settings, as well as patient and provider preferences.

The variation in treatment regimens observed in this analysis may also reflect earlier treatment guidelines within respective cohort collaboration settings. A slightly higher proportion of UK CHIC participants initiated ART between 2004 and 2007 compared with CANOC participants. Treatment guidelines published by BHIVA in 2003 33 and 2005 34 recommended NNRTIs over boosted PIs as the third ART agent, which could account for the comparatively low use of boosted PIs within UK CHIC overall. In contrast, the US Department of Health and Human Services (DHHS) treatment guidelines from 2003 and 2005 recommended both NNRTI‐based regimens and PI‐based regimens as equally acceptable initial ART regimens 35, 36.

### Mortality

According to World Health Organization (WHO) data from 2013, the adult all‐cause mortality rate among those aged 15 to 60 years in Canada is lower than in the UK (0.66 *vs*. 0.72 per 100 000 people, respectively) 37. However, 2011 reports suggest that Canada has a higher HIV/AIDS‐specific mortality rate (1.1 *vs*. 0.8 per 100 000 people, respectively) 38. Our analysis found that all‐cause mortality rates among CANOC and UK CHIC study participants were not significantly different both within the first year following ART initiation and during the entire follow‐up period, after adjusting for confounding variables and accounting for loss to follow‐up as a competing risk.

Limited studies have explored differences in all‐cause mortality rates among individuals living with HIV in Canada and the UK. A recent publication evaluated between‐cohort heterogeneity among participating cohorts within the ART‐CC, and reported a higher all‐cause mortality rate within the North American cohorts compared with European cohorts, even after accounting for perceived completeness of ascertainment of mortality, loss to follow‐up and baseline sociodemographic and clinical characteristics 19. This finding was attributed to the higher proportion of socially marginalized individuals within North American study cohorts, who were incorrectly adjusted for in the analysis as a consequence of missing transmission risk data 19. We attempted to adjust for the higher proportion of socially marginalized participants in the CANOC collaboration by limiting inclusion to participants reporting sexual transmission risk. However, we were unable to control for participant ethnicity, a key marker of socioeconomic variation and access to care within both collaboration settings, due to the large proportion of missing data within the CANOC data set.

In line with our findings, previous work has suggested that differences in sociodemographic and clinical characteristics and health service provision between HIV‐positive cohorts may have less impact on mortality following initiation of modern ART regimens. A collaborative cohort study comparing mortality rates between PLWH in high‐ and low‐income settings observed that, despite baseline differences in sociodemographic, clinical and HIV health service factors, participants demonstrated similar treatment outcomes and mortality rates in the two settings after ART initiation 39.

Additional factors significantly associated with increased all‐cause mortality risk in our analysis included older age at ART initiation and lower CD4 cell count at ART initiation. These findings are consistent with previous mortality analyses within international cohort collaborations 39, 40, 41, 42, and support the consensus that ART should be initiated early in the clinical disease course to optimize clinical outcomes 43, 44. While there appeared to be a nominal decrease in mortality with advancing era of ART initiation in both the CANOC and UK CHIC studies, era of ART initiation did not significantly affect all‐cause mortality in the adjusted competing risks survival analysis. Previous studies in North American 41, European 45 and multinational Euro‐North American settings 40 have reported decreased mortality in modern calendar periods. Our findings may signify that changes in mortality risk by era are attributed to the introduction of modern antiretroviral regimens.

### Limitations

Readers should be aware of several limitations of our analysis. We excluded participants who acquired HIV through IDU from the main analysis to improve cohort comparability. However, MSM who reported IDU as a secondary transmission risk category could not be identified within the UK CHIC study and were coded as MSM, thus were not excluded from the main analysis. Due to the low prevalence of IDU among PLWH in the UK CHIC study, the misclassification is unlikely to have caused a considerable bias in our findings.

Completeness of death ascertainment varied across participating cohorts, with the majority of UK CHIC study mortality data supported by linkages to national vital statistics databases, and weaker vital statistics linkage capabilities overall within the CANOC collaboration. We hypothesize that the weaker ascertainment of death in the CANOC collaboration may contribute to the numerically lower mortality rates seen among CANOC participants in the multivariable analyses. A final limitation was the lack of cause‐specific mortality data within this analysis. As cause‐specific mortality data were not consistently collected across all cohorts, we were unable to differentiate between all‐cause and HIV‐specific mortality in this analysis.

## Conclusions

Among participants enrolled in two longitudinal, multi‐site clinical HIV‐positive cohorts in Canada and the UK who acquired HIV though sexual transmission routes, we observed no statistically significant difference in all‐cause mortality between cohort collaborations, despite varying approaches to clinical care and characteristics of PLWH across the two study settings.

## Author contributions

RSH, CS, HS, AC, SJ and SP conceived the idea for the study. RSH, CS, JA, AB, CC, TH, MH, MK, ML, FM, NM, JM, MN, JR SR and CT contributed to acquisition of data. Statistical analysis was conducted by JZ and ED. Data interpretation was conducted by SP, RH, SJ, JR, ED, HS, JZ and AC. SP, SJ, AC and HS drafted the initial manuscript, and all authors contributed to the final version. All authors have read and approved the final manuscript.

## Research Team Members

The CANOC Collaborative Research Centre: Principal Investigator: Robert Hogg (British Columbia Centre for Excellence in HIV/AIDS; Simon Fraser University); Site Principal Investigators: Ann N. Burchell [Ontario HIV Treatment Network (OHTN); University of Toronto; OHTN Cohort Study (OCS)], Curtis Cooper (University of Ottawa; OCS), Deborah Kelly (Memorial University of Newfoundland), Marina Klein (Montreal Chest Institute Immunodeficiency Service Cohort; McGill University), Mona Loutfy (University of Toronto; Maple Leaf Medical Clinic; OCS), Nima Machouf (Clinique Medicale l'Actuel; Université de Montréal), Julio Montaner (British Columbia Centre for Excellence in HIV/AIDS; University of British Columbia), Janet Raboud (University of Toronto; University Health Network; OCS), Chris Tsoukas (McGill University), Stephen Sanche (University of Saskatchewan), Alexander Wong (University of Saskatchewan); Co‐Principal Investigators: Tony Antoniou (St Michael's Hospital; University of Toronto; Institute for Clinical Evaluative Sciences), Ahmed Bayoumi (St Michael's Hospital; University of Toronto), Mark Hull (British Columbia Centre for Excellence in HIV/AIDS), Bohdan Nosyk (British Columbia Centre for Excellence in HIV/AIDS; Simon Fraser University); Co‐Investigators: Angela Cescon (Northern Ontario School of Medicine), Michelle Cotterchio (Cancer Care Ontario; University of Toronto), Charlie Goldsmith (Simon Fraser University), Silvia Guillemi (British Columbia Centre for Excellence in HIV/AIDS; University of British Columbia), P. Richard Harrigan (British Columbia Centre for Excellence in HIV/AIDS; University of British Columbia), Marianne Harris (St Paul's Hospital), Sean Hosein (Community AIDS Treatment Information Exchange (CATIE)), Sharon Johnston (Bruyère Research Institute; University of Ottawa), Claire Kendall (Bruyère Research Institute; University of Ottawa), Clare Liddy (Bruyère Research Institute; University of Ottawa), Viviane Lima (British Columbia Centre for Excellence in HIV/AIDS; University of British Columbia), David Moore (British Columbia Centre for Excellence in HIV/AIDS; University of British Columbia), Alexis Palmer (British Columbia Centre for Excellence in HIV/AIDS; Simon Fraser University), Sophie Patterson (British Columbia Centre for Excellence in HIV/AIDS; Simon Fraser University), Peter Phillips (British Columbia Centre for Excellence in HIV/AIDS; University of British Columbia), Anita Rachlis (University of Toronto; OCS), Sean B. Rourke (University of Toronto; OCS), Hasina Samji (British Columbia Centre for Excellence in HIV/AIDS), Marek Smieja (McMaster University), Benoit Trottier (Clinique Medicale l'Actuel, Université de Montréal), Mark Wainberg (McGill University; Lady Davis Institute for Medical Research), Sharon Walmsley (University Health Network; University of Toronto); Collaborators: Chris Archibald (Public Health Agency of Canada Centre for Communicable Diseases and Infection Control), Ken Clement (Canadian Aboriginal AIDS Network), Monique Doolittle‐Romas (Canadian AIDS Society), Laurie Edmiston (Canadian Treatment Action Council), Sandra Gardner (OHTN; University of Toronto; OCS), Brian Huskins (Canadian Treatment Action Council), Jerry Lawless (University of Waterloo), Douglas Lee (University Health Network; University of Toronto; Institute for Clinical Evaluative Sciences (ICES)), Renee Masching (Canadian Aboriginal AIDS Network), Stephen Tattle (Canadian Working Group on HIV & Rehabilitation), Alireza Zahirieh (Sunnybrook Health Sciences Centre); Analysts and Staff: Claire Allen (Regina General Hospital), Stryker Calvez (Saskatoon HIV/AIDS Research Endeavour (SHARE)), Guillaume Colley (British Columbia Centre for Excellence in HIV/AIDS), Jason Chia (British Columbia Centre for Excellence in HIV/AIDS), Daniel Corsi (The Ottawa Hospital Immunodeficiency Clinic; Ottawa Hospital Research Institute), Louise Gilbert (Immune Deficiency Treatment Centre), Nada Gataric (British Columbia Centre for Excellence in HIV/AIDS), Lucia Light (OHTN), David Mackie (The Ottawa Hospital), Costa Pexos (McGill University), Susan Shurgold (British Columbia Centre for Excellence in HIV/AIDS), Leah Szadkowski (University Health Network), Chrissi Galanakis (Clinique Médicale L'Actuel), Benita Yip (British Columbia Centre for Excellence in HIV/AIDS), Jaime Younger (University Health Network), and Julia Zhu (British Columbia Centre for Excellence in HIV/AIDS).

The UK CHIC study: Steering Committee: Jonathan Ainsworth, Sris Allan, Jane Anderson, Abdel Babiker, David Chadwick, Valerie Delpech, David Dunn, Martin Fisher*, Brian Gazzard, Richard Gilson, Mark Gompels, Phillip Hay, Teresa Hill, Margaret Johnson, Sophie Jose, Stephen Kegg, Clifford Leen, Fabiola Martin, Mark Nelson, Chloe Orkin, Adrian Palfreeman, Andrew Phillips, Deenan Pillay, Frank Post, Jillian Pritchard, Caroline Sabin, Memory Sachikonye, Achim Schwenk, Anjum Tariq and John Walsh. Central Co‐ordination: University College London (Teresa Hill, Sophie Jose, Andrew Phillips, Caroline Sabin and Alicia Thornton); Medical Research Council Clinical Trials Unit at UCL (MRC CTU at UCL), London (David Dunn and Adam Glabay). Participating Centres: Brighton and Sussex University Hospitals NHS Trust (Martin Fisher*, Nicky Perry, Stuart Tilbury, Elaney Youssef and Duncan Churchill); Chelsea and Westminster Hospital NHS Foundation Trust, London (Brian Gazzard, Mark Nelson, Rhiannon Everett, David Asboe and Sundhiya Mandalia); King's College Hospital NHS Foundation Trust, London (Frank Post, Hardik Korat, Chris Taylor, Zachary Gleisner, Fowzia Ibrahim and Lucy Campbell); Mortimer Market Centre, UCL, London (Richard Gilson, Nataliya Brima and Ian Williams); Royal Free NHS Foundation Trust/UCL, London (Margaret Johnson, Mike Youle, Fiona Lampe, Colette Smith, Rob Tsintas, Clinton Chaloner, Samantha Hutchinson, Caroline Sabin, Andrew Phillips, Teresa Hill, Sophie Jose, Alicia Thornton and Susie Huntington); Imperial College Healthcare NHS Trust, London (John Walsh, Nicky Mackie, Alan Winston, Jonathan Weber, Farhan Ramzan and Mark Carder); Barts and The London NHS Trust, London (Chloe Orkin, Janet Lynch, James Hand and Carl de Souza); Homerton University Hospital NHS Trust, London (Jane Anderson and Sajid Munshi); North Middlesex University Hospital NHS Trust, London (Jonathan Ainsworth, Achim Schwenk, Sheila Miller and Chris Wood); The Lothian University Hospitals NHS Trust, Edinburgh (Clifford Leen, Alan Wilson and Sheila Morris); North Bristol NHS Trust, Bristol (Mark Gompels and Sue Allan); University Hospitals of Leicester NHS Trust, Leicester (Adrian Palfreeman, Khurram Memon and Adam Lewszuk); South Tees Hospitals NHS Foundation Trust, Middlesbrough (David Chadwick, Emma Cope and Jane Gibson); Lewisham and Greenwich NHS Trust, Woolwich, London (Stephen Kegg, Paul Main, Dr Susan Mitchell and Dr Meg Hunter); St George's Healthcare NHS Trust, London (Phillip Hay and Mandip Dhillon); York Teaching Hospital NHS Foundation Trust, York (Fabiola Martin and Sarah Russell‐Sharpe); University Hospitals Coventry and Warwickshire NHS Trust, Coventry (Sris Allan, Andrew Harte and Stephen Clay); The Royal Wolverhampton Hospitals NHS Trust, Wolverhampton (Anjum Tariq, Hazel Spencer and Ron Jones); Ashford and St Peter's Hospitals NHS Foundation Trust, Chertsey (Jillian Pritchard, Shirley Cumming and Claire Atkinson); Public Health England, London (Valerie Delpech); UK Community Advisory Board (Roy Trevelion). *Deceased.
